# High value and low-cost virtual reality healthcare professional education: proceedings of a roundtable workshop

**DOI:** 10.15694/mep.2020.000057.1

**Published:** 2020-03-30

**Authors:** Kieran Walsh, Mohamed Elhassan Abdalla, Pasquale Berlingieri, Jonathan Foo, Dragan Ilic, Stephen Maloney, Jack Pottle, George Rivers

**Affiliations:** 1 BMJ; 2 College of Medicine and Medical Education Center; 3 Division of Surgery & Interventional Science; 4 Department of Physiotherapy; 5 Medical Education Research & Quality (MERQ) unit Public Health and Preventive Medicine Monash University; 6 Oxford Medical Simulation; 7 Department of Economics Monash Business School Monash University

**Keywords:** Value, cost, virtual reality, healthcare professional education

## Abstract

This article was migrated. The article was marked as recommended.

Introduction

The following represents proceedings of an online roundtable workshop on high value and low-cost virtual reality healthcare professional education. The purpose of the workshop was to seek the views of professionals from different sectors and different countries on moving toward high value and low-cost virtual reality education. The workshop was comprised of eight delegates with an interest in this subject. The delegates were from Europe, the Middle East, and Australia. They represented different sectors - including clinical medicine, educational economics, health professional education, simulation, technology, evidence-based methodologies, and industry.

Themes

The following themes emerged from the workshop: the challenge of thinking about the cost of virtual reality from the points of view of the payer and the user; the core need to define the context of use of virtual reality; the absolute need to define the purpose of a virtual reality programme; the recognition of the growing opportunity of multiplayer virtual reality; the need to exploit the unique properties of virtual reality; the importance of realising that there are already various forms of virtual reality available and these can achieve different outcomes at different costs; the need to integrate virtual reality into the rest of the curriculum; and the various forms of cost analysis that might be suitable for evaluating the cost and outcomes of virtual reality.

Conclusions

In the long-term, a growing body of evidence that is based on original research and systematic reviews will help us decide what is high value and low-cost virtual reality in healthcare professional education. However, a strategic approach is needed to ensure that the original research concentrates on the right topics that will yield the most value to education decision-makers and to other related stakeholders. We feel that non-hierarchical interdisciplinary roundtable discussions are an effective means of planning strategy.

## Introduction

The following represents proceedings of an online roundtable workshop on high value and low-cost virtual reality healthcare professional education. The purpose of the workshop was to seek the views of professionals from different sectors and different countries on moving toward high value and low-cost virtual reality education. The goal of this article is to inform the research and implementation priorities for taking this field forward.

Virtual reality may be defined as an artificial environment that is created with software and presented to the user in such a way that the user suspends belief and accepts it as a real environment" (Whatis.com, n.d. https://whatis.techtarget.com/definition/virtual-reality-gaming-VR-gaming). It uses “real-time interactive graphics with 3D models, combined with a display technology that gives the user the immersion in the model world and direct manipulation” ( Fuchs and Bishop, 1992).

Virtual reality involves use of technology to deliver a learning objective, and is increasingly used in healthcare professional education ( Bernardo, 2017; Pottle, 2019). Like all forms of healthcare professional education, virtual reality is associated with costs ( Maloney *et al.,* 2017).

## The workshop

The workshop was comprised of eight delegates with an interest in this subject. The delegates were from Europe, the Middle East, and Australia. They represented different sectors - including clinical medicine, educational economics, health professional education, simulation, technology, evidence-based methodologies, and industry. The group was purposely chosen to ensure that multiple perspectives were captured in the discussion.

All the delegates at the roundtable contributed to and are authors of this paper. All took part in discussions and had the opportunity to add to or refute any of the comments that were made.

At the start of the workshop, all delegates were invited to suggest their priorities for driving forward the research and implementation agendas in this field. A wider discussion was then opened up. The following themes emerged from this discussion.

## Themes

Cost of virtual reality from different perspectives

The first theme to emerge was the challenge of thinking about the cost and value of virtual reality from the points of view of the payer and the user. Sometimes the payer and the user will be the same - if it is an individual purchaser for individual use. With respect to undergraduate education, the payer will often be the institution - for example a medical school - and here the users will be medical students. The payer will most likely value scaling up use and ensuring implementation so that as many learners as possible use the virtual reality. A medical school might wish to scale up educational provision and address workforce shortages - and use virtual reality as one of many means to achieve this. But the medical student might value different aspects of virtual reality. They might be thinking about their individual career plan and how virtual reality could help them develop certain skills - for example, dexterity in laparoscopic surgery. Users might enjoy experimenting with new technologies and value virtual reality for this reason. Some might simply like “playing” on virtual reality-based simulations (which of itself might be a good outcome - with enjoyment can come engagement and learning - although enjoyment should not be seen as a sole intended outcome in and of itself). Ideally outcomes would be aligned - the payer and the user wanting to achieve similar outcomes.

The ability for virtual reality to achieve value may be compromised if there’s ‘tension’ between what the payer and the user intend to achieve. The dilemma of needing to satisfy the payer and the user is a challenge in many areas of healthcare professional education, but there may be particular problems with virtual reality. Payers and users may have different perspectives on technological innovations. Some payers may not use virtual reality themselves, and therefore wonder why anyone should. Some may dismiss the concept as “people playing video games”. As a result, some payers may look for the lowest cost solutions available. Similar outcomes may result when the payers feel that they are not sufficiently technologically competent to test the programme. However, it is incumbent on industry and institutions to make sure that this does not become an insurmountable problem.

“It needs to make sense from a cost and value perspective from everyone in the chain. Payers are usually more focussed on cost and less aware of value. Educators - in the middle - have a good split of cost and value. Students only care about value - they don’t care about cost - because they don’t necessarily get to see the costs”

### Context of use of virtual reality

The second theme related to the core need to define the context of use of virtual reality. What might be low cost high value virtual reality in one context might be high cost and low value in another. For example, virtual reality can be used in undergraduate education, postgraduate education, and continuous professional development. However, users from each of these stakeholder groups will have different priorities. If undergraduates are the payers, then they will likely value low cost or free virtual reality that will help them pass exams (such as Objective Structured Clinical Examinations). However, if their educational institution is the payer, then the institutional payer will more likely value quality, accessibility and alignment with their examination and clinical objectives.

In postgraduate education, a competing priority is time and scheduling - how will the user have time to learn and also to work? This real-world problem needs to be addressed to ensure accessibility and thus implementation. Other priorities in postgraduate education are clinical utility, quality, and flexibility. This is all dependent on knowing the target audience. Sometimes the different priorities of different groups are conflicting, but as often as not, they act as a corollary to each other (as in this case).

### Defining the purpose of virtual reality programmes

The third theme was the absolute need to define the purpose of a virtual reality programme. Without clarity of purpose, it will be impossible to know if value has been achieved. Some virtual reality training programmes have very specific aims - for example to shorten the initial phases of the learning curve before the trainee surgeon goes to theatre. This aim is specific and measurable - in terms of time (and from an economic perspective, time is money). However, some virtual reality programmes, or new technologies, do not have clear aims and sometimes appear to want to solve a problem that doesn’t exist in the first place. There are many examples of technologies that were popular and then faded (for example Second Life or Google Glass). Clarity of purpose is essential - but that is not to say that virtual reality needs to have a single purpose. In fact, virtual reality can have multiple primary and even secondary objectives. For example, virtual reality can be used to evaluate competency in both formative and summative assessments, such as OSCEs. It may be that virtual reality could save considerable resources in OSCEs - as it might save the expenses associated with the logistics of having a physical station ( Brown *et al.,* 2015).

Likewise, its capability of recording activity logs and standardised responses may avert significant cost-consequences such as legal challenges to high-stakes assessment results. The evidence base in this field would need to be developed and any new forms of assessment evaluated for reliability and validity, but there was consensus in the group that high cost forms of education or assessment were ripe areas for research - as they might be associated with the greatest opportunity for the savings. They might also be associated with the most value - with virtual reality enabling outcomes associated with “assessment for learning” as opposed to “assessment of learning” - and so virtual reality enabling the exam candidate to review their performance on the screen.

“It all comes back to your “why”. That is, what are your trying to achieve with using virtual reality. In some cases, you do need high fidelity with haptic feedback because you are trying to teach a physical task. In other cases, if it is about the environment that you are trying to teach, then you might not need haptic feedback. So, it comes back to what is your why and aligning that with the solution that you pick.”

### Multiplayer virtual reality

The fourth theme was the recognition of the growing opportunity of multiplayer virtual reality. Multiplayer virtual reality might enable important outcomes such as interprofessional education and better interpersonal team working skills. Gaming technology certainly shows the popularity of this technology with the next generation of learners. Multiplayer programmes have by their nature greater fidelity to the real-life environment - as real life involves other people so allowing for more interesting and unpredictable outcomes. Multiplayer programmes could also enable provision to be scaled up and thus to generate economies of scale - with the same technology being used by far greater numbers of users. Multiplayer programmes are also important if one of the key objectives of the intervention is to maximise uptake and synchronisation - where value is increased exponentially as more nodes (players) are added to the player network. The concept of multiplayer programmes moves virtual reality in a very different direction to those who might simply be tempted to replace faculty with a tool. This educational strategy can affect the learner’s behaviour whose enthusiasm may fade away in the absence of human instructor feedback ( Mahmood and Darzi, 2004).

However, if one of the players in a multiplayer virtual reality tool is a facilitator, tutor, expert observer or debriefer, then virtual reality can become a means to amplify the work of the teacher.

The mission of an educator is to value feedback and to educate based on feedback - which is an ongoing process helping the trainee to build on a foundation of skills and behaviours. Virtual reality should be able to help with this process. One final caveat regarding the use of multiplayer programmes is that some junior learners will still want single player programmes - as they don’t want people to see their mistakes.

“The multiplayer idea - maybe that is the high value that virtual reality brings that other tools cannot.”

### Exploiting the unique properties of virtual reality

The fifth theme to emerge related to ensuring that virtual reality is not wasted. This is by making sure that the properties that are unique to virtual reality are exploited to maximum effect. Virtual reality can be used for many purposes - such as learning how to perform laparoscopic surgery - where the focus should be on an immersive and interactive experience. It is not the best use of virtual reality to create programmes on common clinical topics like how to do an abdominal examination. This is because the haptics required to do this are not yet sufficiently sophisticated, and also because it is probably easier as well as less expensive for the student to go to the ward and examine real patients. Despite such limitations, virtual reality can enable learners to practice skills that they will not see every day - such as auscultation for the fine crackles heard in fibrotic lungs or running a cardiac arrest in a young patient with hypothermia, or dealing with dangerous situations (for example, dealing with violent patients). It is the task of educators to distil virtual reality down to the essential parts that really work, ensuring it is fit for purpose, and from which all can reap maximum value.

### Different forms of virtual reality

The sixth theme raised was the various forms of virtual reality that are already available and how these can achieve different outcomes at different costs. Virtual reality can use state-of-the-art technology that results in a fully immersive experience. This might be of value in some circumstances but will not be necessary in all. The value in virtual reality is making learners believe that they are in a real situation. So, educators should think about using technology that will make them believe that they are in a real world. Virtual reality headsets are now much more widely available, and it might feel more natural for people to use their own equipment. This should also ensure that virtual reality is actually used, and not just stored in a corner of the room in a simulation centre.

This strategy has an additional effect of decentralising at least a part the cost to the end users. However, it is still only part of the cost. It is best practice to think of hardware and software costs as separate. Hardware is a variable cost, but software can be a fixed cost - and this is where economies of scale can be achieved.

“On the hardware side, if we can strive for hardware that is readily available. And on the software side, having a platform from which you can achieve economies of scale”

### Virtual reality in the curriculum

The seventh theme was how virtual reality might relate to the rest of the curriculum that the learner must work through. Virtual reality can be complementary to physical simulation or to learning in the workplace, or it can be used as a stepping stone to support learners in the transition to physical simulation. This blended approach might be both effective and offer optimal value. Virtual reality that is part of a curriculum clearly becomes virtual reality that is made available through an institution. If curricula are national, then institutions should consider setting up consortia to achieve value by means of a group purchase (essentially buying in bulk and also purchasing group insurance for maintenance).

### Forms of cost analysis

The eighth theme was the type of cost analysis that might be undertaken in the field of virtual reality. At its simplest, a cost accounting analysis might be used to evaluate costs from the perspective of the payer - for budgeting and return on investment purposes. More broadly, full economic evaluations might look at the costs of virtual reality, the positive outcomes achieved, and the negative outcomes avoided, from the perspective of multiple stakeholders. This would take virtual reality out of a limited financial accounting framework into a societal economic framework. This is not as straightforward but might yield much deeper and richer insights.

## Concluding remarks

The themes in this discussion on the cost and value of virtual reality in healthcare professional education included: the perspectives of different stakeholders; the context of use; the core purpose of virtual reality; the different forms of virtual reality already available and the growing importance of multiplayer virtual reality; the exploitation of virtual reality to maximum effect; curriculum integration; and cost analyses that might be appropriate to virtual reality. It should be noted that, even though many of the examples in this paper relate to surgical simulation, virtual reality can be used in a variety of contexts and specialities (spanning from internal medicine to obstetrics, including nursing and midwifery).

We are still likely to be in the early stages of virtual reality in healthcare professional education. This field of education will change rapidly over the coming years and there will be multiple drivers of this change. Cost will be one driver of change. Another key driver will be the perspective of the end users, and this will change over time. The end users might currently be undergraduate students or postgraduate trainees - but in a few years they will become educators, purchasers and commissioners. As learners who have grown up with virtual reality, they will have a completely different perspective to their predecessors. Their new perspectives will have considerable influence on their decisions and on the next generation of learners.

In the long-term, a growing body of evidence that is based on original research and systematic reviews will help us decide what is high value and low-cost virtual reality in healthcare professional education. However, a strategic approach is needed to ensure that the original research concentrates on the right topics that will yield the most value to education decision-makers and to other related stakeholders. We feel that non-hierarchical interdisciplinary roundtable discussions are an effective means of planning strategy.

Our group plans to use the outcomes of this workshop to decide our next steps in researching and implementing virtual reality programmes so that all stakeholders can achieve maximum value from this emerging modality. We feel that a long term, multi-perspective and strategic approach is needed to ensure that virtual reality delivers value for the benefit of both patients and populations alike.

## Take Home Messages

• Virtual reality involves use of technology to deliver a learning objective, and is increasingly used in healthcare professional education.
• Like all forms of healthcare professional education, virtual reality is associated with costs (costs in this article refer to financial costs).
• Ideally virtual reality healthcare professional education would be high value and low-cost.

## Notes On Contributors

**Kieran Walsh** works at the BMJ Knowledge Centre, BMJ, London.

**Mohamed Elhassan Abdalla** works at the College of Medicine and Medical Education Center, University of Sharjah, UAE.

**Pasquale Berlingieri** works at the Division of Surgery & Interventional Science, Royal Free Campus, UCL, London, UK: Centre for Screen-Based Medical Simulation, Royal Free Hospital, London, UK.

**Jonathan Foo** works at the Department of Physiotherapy, Faculty of Medicine, Nursing, and Health Sciences, Monash University, Australia.

**Dragan Ilic** works at the Medical Education Research & Quality (MERQ) unit, Public Health and Preventive Medicine, Monash University.

**Stephen Maloney** works at the Department of Physiotherapy, Faculty of Medicine, Nursing, and Health Sciences, Monash University, Australia.

**Jack Pottle** works at Oxford Medical Simulation, London, UK.

**George Rivers** works at the Department of Economics, Monash Business School, Monash University, Australia.

## Declarations

The author has declared the conflicts of interest below.

KW works for BMJ which produces a range of resources in technology enhanced learning for healthcare professionals. JP works for Oxford Medical Simulation which produces virtual reality simulation for the education and assessment of healthcare professionals.

## Ethics Statement

This was not a research study. It is a report of a roundtable meeting.

## External Funding

This article has not had any External Funding
