# Supplementary material for: High value and low-cost virtual reality healthcare professional education: proceedings of a roundtable workshop
Source: MedEdPublish (2016). 2020 Mar 30;9:57. [Version 1] doi: 10.15694/mep.2020.000057.1 (PMC10697468; doi:10.15694/mep.2020.000057.1)
Supplement: Peer review history

# Reviewer report: Reviewer response for version 1

**DOI:** 10.21956/mep.19992.r30430

**Published:** 2020-04-08

**Authors:** P Ravi Shankar

**Affiliations:** 1 American International Medical University

This review has been migrated. The reviewer awarded 3 stars out of 5 This is an interesting article on the use of virtual reality in medical education. The round table discussed the use of high value and low cost virtual reality. The paper is well-written and the authors put forward a number of themes and issues. They also put forward some ideas to reduce the cost of the resources. The hardware associate with virtual reality is becoming more widely available and cheaper as mentioned by the authors. Some of the features like haptics may be more expensive. Many content providers offer a yearly payment plan based on the number of students in the institution and the intended use of the resources. The authors have primarily concentrated on surgical simulation. One of the problems is the terms low cost and high value have not been defined by the authors. I do understand that both these have a significant user perspective but some general benchmarking may be useful. A description of the process followed during the workshop and used to derive the themes would be useful. The participants were primarily from institutions in high income countries. I believe their recommendations especially on the cost will have to be critically examined by educators in more resource limited settings. The authors can add a limitations section mentioning some of the limitations of the workshop which can impact on the generalizability of their recommendations.

Reviewer Expertise:

NA

No decision status is available

# Reviewer report: Reviewer response for version 1

**DOI:** 10.21956/mep.19992.r30429

**Published:** 2020-04-01

**Authors:** Deb Halder

This review has been migrated. The reviewer awarded 3 stars out of 5 The paper tended to define ‘virtual reality’ which is appreciated but the in-text referencing ‘(Whatis.com, n.d. https://whatis.techtarget.com/definition/virtual-reality-gaming-VR-gaming)’ loses the academic appearance of the insertion. Again, the introduction could have explained what ‘high –value’ and ‘low cost’ really means. The introduction cannot provide the problems of the paper and what it tends to seek along with its defined objectives. Again, this idea ‘Like all forms of healthcare professional education, virtual reality is associated with costs (Maloney et al., 2017)’ cannot be understood what it wants to present as it ended the introduction.The title under ‘Workshop’ aimed at providing background of the case. But it could not finish providing details of what was really targeted and if there was fixed questionnaire or guiding procedures of that workshop. Again, there are eight more themes discussed. But, the discussion has turned to be mere story telling. The authors should have added references to defend their conceptions and terminologies. A lot of things are interlinked within an academic publishable paper. It claims logical construction of the body and reliable sources of references to make the supposition valid.The conclusion has created a diversion from the above discussions. This part could have summed up; rather it has gone on detail expression of prospects of virtual reality. Authors cannot prospect if it is not evaluated and described at the main body of the paper.

Reviewer Expertise:

NA

No decision status is available

# Reviewer report: Reviewer response for version 1

**DOI:** 10.21956/mep.19992.r30427

**Published:** 2020-03-31

**Authors:** Puja Dulloo

**Affiliations:** 1 Pramukhswami Medical College

This review has been migrated. The reviewer awarded 5 stars out of 5 I enjoyed reading this article, as this is what in medical educational set-up we need to explore. Starting with the first theme, "cost of virtual reality from different perspectives", which highlights who would focus on the value of the VR and who would be inclined to look on the cost. Although, I strongly believe that a postgraduate value the VR purpose as well as is concerned about its cost, so will use it to utmost. We can explain the importance of VR for learning to undergraduate students. The third theme, "defining the purpose of virtual reality programmes", really was good one which could be well connected with the last theme of integrating it within the curriculum. Once we know the purpose of the VR then it will be well used for teaching learning and subsequently the assessment, which is explained in second theme i.e., "Context of use of virtual reality". Use of VR globally, during this pandemic state, is turning out to be a better solution during present time. One aspect which we should not forget is to sensitize the users i.e., the undergraduate students. Otherwise the cost will be higher due to less usage of the VR. While planning this study, I think, an undergraduate student should have been a part of the workshop.Thanks to the entire panelist to for sharing their thoughts.

Reviewer Expertise:

NA

No decision status is available

# Reviewer report: Reviewer response for version 1

**DOI:** 10.21956/mep.19992.r30428

**Published:** 2020-03-30

**Authors:** Trudie Roberts

**Affiliations:** 1 Leeds Institute of Medical Education

This review has been migrated. The reviewer awarded 5 stars out of 5 I enjoyed reading this submission and it helped me crystallise my own thinking in the area of VR. It was nice to see 'defining the purpose" as one theme although personally I would have moved it up to number 1. The seventh theme VR in the curriculum is also vitally important. If VR is not integrally linked to the curriculum it will be seen as a bolt on extra which can be ditched in times of austerity. However VR is an important tool in delivering those areas of the curriculum which are historically challenging for example emergency situations. It is not however an end in itself.The development of multiplayer VR very exciting and it has huge potential.The obvious use of VR to support education in challenging times like the Covid 19 pandemic is very apposite.Thank you to the panel for sharing their thoughts and discussion.

Reviewer Expertise:

NA

No decision status is available
